# The Development and Validation of an LC-MS/MS Method for the Determination of Glyphosate, AMPA, and Glufosinate in Honey Following FMOC-Cl Derivatization: Application to Italian Samples

**DOI:** 10.3390/foods14234050

**Published:** 2025-11-26

**Authors:** Marianna Martinello, Sara Zanella, Franco Mutinelli, Michela Bertola

**Affiliations:** 1 NRL for Honey Bee Health, Istituto Zooprofilattico Sperimentale delle Venezie, Viale dell’Università 10, 35020 Legnaro, Italy; fmutinelli@izsvenezie.it (F.M.); mbertola@izsvenezie.it (M.B.); 2 Department of Agronomy, Food, Natural Resources, Animals and Environment, University of Padua, Viale dell’Università 16, 35020 Legnaro, Italy; sara.zanella.3@phd.unipd.it

**Keywords:** honey, glyphosate, AMPA, glufosinate-ammonium, FMOC-Cl, LC-MS/MS, validation, monitoring

## Abstract

Glyphosate-based herbicides are among the most widely used pesticides worldwide, but data on their occurrence in food products, particularly honey, remain limited. The analytical determination of glyphosate, its primary metabolite aminomethylphosphonic acid (AMPA), and glufosinate ammonium is technically challenging due to their high polarity and distinctive physicochemical properties, requiring the development of dedicated single-residue analytic methods. In this study, honey samples were prepared by derivatizing the target analytes with 9-fluorenylmethyl chloroformate (FMOC-Cl), followed by solid-phase extraction (SPE) using hydrophilic–lipophilic balanced (HLB) cartridges to improve matrix clean-up and enhance analytical sensitivity. Quantification was performed by liquid chromatography coupled with tandem mass spectrometry (LC-MS/MS). The optimized method was validated according to the SANTE/11312/2021 guidelines, with all parameters, including sensitivity, linearity, mean recovery (accuracy), precision (RSDr), and limit of quantification (LOQ), meeting the required performance criteria. The validated method was applied to 126 honey samples of various botanical origins, representative of Italian production. The results indicated a frequent detection of glyphosate residues, although concentrations were generally low and remain below levels of regulatory concern.

## 1. Introduction

The decline of insect pollinators is a global concern, and pesticide use has been identified as one of its potential drivers. Glyphosate-based herbicides (GBHs) are the most widely used pesticides worldwide; however, until recent years, they were generally considered safe for non-target organisms such as pollinators [1]. Glyphosate, a synthetic organophosphorus herbicide, has been extensively used in agriculture for weed control since its commercial introduction in the 1970s. Its broad-spectrum efficacy and cost-effectiveness have made it a key component of modern crop production systems, particularly following the development of genetically modified crops engineered for glyphosate resistance. According to Pesticide Action Network (PAN) Europe, glyphosate global use has increased nearly 15-fold since the introduction of glyphosate-tolerant genetically engineered crops in 1996. In 2012, Europe accounted for approximately 16.6% of global glyphosate use, and by 2017, glyphosate represented 33% of the total herbicide market within the European Union (EU) [2]. Despite its widespread application, glyphosate has raised considerable concerns regarding potential impacts on human health and environmental safety. In 2015, the International Agency for Research on Cancer (IARC) classified glyphosate as “probably carcinogenic to humans” (Group 2A), based on limited evidence linking exposure to an increased risk of non-Hodgkin lymphoma. This classification prompted intense international debate over glyphosate’s safety and regulatory status. However, subsequent assessment by the European Food Safety Authority (EFSA) and the United States Environmental Protection Agency (EPA) concluded that glyphosate is unlikely to pose a carcinogenic risk to humans [3,4]. In November 2023, the EU renewed the approval of glyphosate for an additional ten years, introducing new conditions and restrictions in response to persistent concerns among researchers and policymakers regarding its potential environmental and health effects. These measures include a ban on pre-harvest desiccation, mandatory protective measures to safeguard non-target organisms, and the possibility for individual Member States to implement stricter national restrictions [5]. For example, Italy prohibits pre-harvest applications (desiccation) and bans glyphosate use in public areas such as parks, playgrounds, and schools or healthcare facilities, as well as on sandy soils to protect groundwater [6]. France has banned glyphosate use by private individuals and in public spaces [7], while Germany plans a full ban by the end of 2025, having already restricted its use in non-agricultural areas [8]. Outside the EU, Luxembourg and Mexico have attempted complete bans, while Vietnam has already implemented one [9]. These initiatives reflect the ongoing global effort to balance agricultural productivity with environmental and public health protection.

Substances that pose particularly high levels of acute or chronic risk to health or the environment are classified as “Highly Hazardous Pesticides”. The list, compiled by the Food and Agriculture Organization of the United Nations (FAO) and the World Health Organization (WHO), includes both glyphosate and glufosinate.

Glufosinate ammonium is a non-selective, broad-spectrum herbicide that has been banned in the EU since 2018, following its classification as toxic to reproduction (Category 1B) under Regulation (EC) No 1107/2009 [10]. Despite its prohibition in Europe, glufosinate remains widely used in North America, particularly in the United States and Canada, where agriculture is dominated by genetically modified crops engineered for herbicide tolerance [11]. Its use is also prevalent across the Asia–Pacific region, with major markets including China, India, and South Korea, driven by the intensification of agricultural production and the adoption of modern farming technologies [12]. In December 2024, the European Commission (EC) submitted a formal request to EFSA to initiate a call for data aimed at addressing toxicological and residue-related data gaps for glufosinate. The objective of this initiative was to support an updated risk assessment of maximum residue levels (MRLs) for glufosinate in accordance with Article 43 of Regulation (EC) No 396/2005 [13,14].

The honey bee *Apis mellifera* (Linnaeus, 1758) is considered a sentinel species within the pollinator community due to its wide foraging range and high sensitivity to environmental contaminants. Among the various agrochemicals to which bees are exposed, glyphosate has been the most widely used pesticide globally over the past five decades [1]. Following its application in agricultural settings, glyphosate residues have been detected in floral resources visited by honey bees and in hive products such as honey, pollen, and wax [15]. These residues can be transferred into the hive, potentially affecting bee health through acute or chronic exposure. Several studies have investigated the toxicological effects of glyphosate on honey bees, revealing both lethal and sublethal impacts. Chronic exposure has been shown to be particularly harmful, affecting both adult bees and larvae by impairing cognitive functions, altering sleep behavior, and reducing foraging efficiency [16]. Exposure to environmentally relevant concentrations of glyphosate has been reported to impair honey bee cognition, resulting in longer flight durations and decreasing foraging success, with potential long-term consequences for colony health [17,18]. Field-relevant concentrations, quantified at approximately 70 glyphosate acid equivalents per liter, based on literature values, have been shown to delay larval development and disrupt gut microbiota, leading to teratogenic effects and increased mortality in newly emerged adults [19]. Moreover, glyphosate-induced dysbiosis reduces the abundance of beneficial gut bacteria, increasing susceptibility to opportunistic infections [20]. Together, these findings suggest a dose-dependent and context-specific toxicological response. Similarly, the effects of glufosinate on honey bees have also been investigated, demonstrating that glufosinate exposure can alter the gut microbiota composition, affect immune system regulation, and reduce survival rates in a dose-dependent and exposure duration-dependent manner, suggesting that even low levels of glufosinate can impair bee health over time [21].

A recent review provided a comprehensive overview of the presence of glyphosate, glufosinate, and their metabolites in honey bee products across multiple countries. Their presence has been confirmed in several hive matrices, although prevalence varies widely. To date, twenty studies have investigated glyphosate residues in honey and related products, detecting the compound in 32% of samples. In most cases, concentrations were below the MRL established by the EU (0.05 mg/kg), although some samples exceeded this threshold. Contamination has also been reported in other hive matrices, including pollen and beeswax. In contrast, glufosinate has been less extensively studied, with only four publications addressing its occurrence in honey. It was detected in only one of these studies, with concentrations ranging from 1 µg/kg to 33 µg/kg [15].

To ensure compliance with MRLs and to assess consumer exposure to pesticide residues in food of both plant and animal origin, the EU implements Multiannual Control Programs for Pesticide Residues (MACPs) on an annual basis. In addition to these EU-wide programs, each Member State conducts its own national control programs (PCNs), which complement the MACPs. To assist Member States in designing their national monitoring programs and to provide guidance on potential future inclusions in MACPs, the EC annually issues a non-binding Pesticides Working Document (WD). Annex VII of the WD lists substances of interest for honey analysis within the PCNs, in accordance with EFSA’s recommendation, to analyze honey samples for substances included in the MACP using the broadest possible analytical scope. This coordinated approach aims to estimate bee exposure and to support the review and adjustment of MRLs for honey. Since 2018, glyphosate has consistently appeared in the Commission’s WD as one of the most frequently detected residues in honey [22].

The determination of glyphosate, AMPA, and glufosinate in honey is particularly challenging due to their high polarity and specific physicochemical properties. Despite the importance of monitoring these compounds for food safety and regulatory compliance, there remains a shortage of validated, sensitive, and broadly applicable analytical methods for their determination in honey under routine laboratory conditions. Recent studies have emphasized the complexity of honey matrices and the necessity for robust approaches, such as liquid chromatography–tandem mass spectrometry (LC-MS/MS) and ion chromatography coupled with high-resolution mass spectrometry (IC-MS/MS), to achieve accurate quantification and to comply with international performance requirements [23,24,25]. The present study addresses this analytical gap by developing and validating a method specifically designed for the simultaneous detection of glyphosate, AMPA, and glufosinate in honey utilizing LC-MS/MS, ensuring suitability for routine monitoring and compliance with food safety standards. The validated method was subsequently applied to a large-scale monitoring campaign involving honey samples collected from multiple regions across Italy, with the aim of assessing the occurrence of glyphosate, AMPA, and glufosinate residues and contributing to support food safety surveillance efforts in alignment with current EU regulatory recommendations.

## 2. Materials and Methods

### 2.1. Chemicals and Reagents

Aminomethylphosphonic acid (AMPA, 100.0% purity), glufosinate ammonium (99.5%), and glyphosate solution 1000 μg/mL in water (99.0%) were purchased from Supelco^®^ (Merck KGaA, Darmstadt, Germany). Glyphosate-FMOC 100 μg/mL in ACN/water (99.6%), AMPA-FMOC 100 μg/mL in ACN/Dimethyl sulfoxide (100.2%), and glufosinate-FMOC (96.9%) were obtained from Dr. Ehrenstorfer™ (LGC Standards, Manchester, UK). Glyphosate-2-^13^C,^15^N (99.9%) was purchased from Supelco^®^, 13C-15N-AMPA solution 100 µg/mL in water (100.0%) and glufosinate-D_3_ solution 100 µg/mL in water (96.5%) were supplied by HPC Standards GmbH (Borsdorf, Germany).

Methanol LC-MS grade and acetonitrile (ACN) LC-MS grade were purchased from Supelco^®^ (Merck KGaA, Darmstadt, Germany). Formic acid (HCOOH) LC-MS grade was obtained from Chem-Lab ANALYTICAL bvba^®^ (Zedelgem, Belgium); ammonium formate (≥99.0%), potassium hydroxide (KOH) ACS reagent (≥85%) pellets, sodium carbonate powder (Na_2_CO_3_) ACS reagent, dimethyl sulfoxide (DMSO) ACS grade, and fluorenylmethylchloroformate (FMOC-Cl, 97%) were purchased from Sigma-Aldrich^®^ (Merck KGaA, Darmstadt, Germany). Dichloromethane (DCM) was obtained from Carlo Erba Reagents S.r.l. (Milan, Italy). Supel™ Swift HLB SPE Tubes (60 mg) were purchased from Supelco^®^. Ultrapure water was produced using an Arium^®^ Pro purification system (Sartorius, Göttingen, Germany).

### 2.2. Standard Solutions

Reference material solutions of AMPA and glufosinate ammonium were prepared at concentrations of 1000 and 100 µg/mL, respectively, in water containing 10% ACN. The dissolution of the powdered standards was facilitated by the addition of a few drops of DMSO. Working mix standard solutions containing glyphosate, AMPA, and glufosinate ammonium were prepared at final concentrations of 10 µg/mL and 1 µg/mL in water with 10% ACN. A corresponding working mix internal standard solution was prepared at the final concentration of 10 µg/mL in water with 10% ACN.

FMOC-derivatized standard solutions of glyphosate, AMPA, and glufosinate were prepared separately in an ACN/water mixture (8:2, *v*/*v*) at a final concentration of 0.1 µg/mL. 

All diluted solutions were stored under appropriate conditions and considered stable for up to six months from the date of preparation or opening. Glyphosate-containing solutions were stored in polyethylene or other plastic containers, protected from light, and refrigerated to maintain stability.

### 2.3. Sample Preparation

Briefly, 2.00 g of thoroughly homogenized honey samples (acacia, *Robinia pseudoacacia*, multifloral, and chestnut, *Castanea sativa*) were accurately weighed into a centrifuge tube, spiked with 50 µg/kg of working mix internal standard solution, and dissolved in 5 mL of an aqueous solution containing 1% formic acid (*v*/*v*). The mixture was sonicated for 10 min to facilitate dissolution and then vortex-mixed for 10 min. The pH of the resulting solution was then adjusted to 7.0 ± 0.5 using 6 N KOH, with fine adjustments made using 0.6 M and 0.1 M KOH solutions as required. An aliquot of 0.5 mL of the solution was transferred into a 2 mL micro-centrifuge tube, followed by the addition of 0.5 mL of 0.1 M sodium carbonate and 0.2 mL of freshly prepared FMOC-Cl (50 mg/mL in ACN). The reaction mixture was vigorously homogenized for 3 min using an automated mixer, then rotary-shaken in the dark for 1 h. Derivatization was quenched by adding 0.32 mL of water containing 1% formic acid, and the sample was centrifuged at 10,000× *g* for 10 min at room temperature.

The supernatant was loaded onto HLB SPE cartridges preconditioned with 6 mL of methanol and 6 mL of water containing 1% formic acid. The columns were washed sequentially with 5 mL of water and 5 mL of DCM, and then dried under vacuum. Elution was performed with 5 mL of methanol, and the eluate was evaporated to dryness under vacuum at 45 °C. The dry residue was reconstituted in 0.5 mL of a 50:50 mixture of mobile phases A and B, where mobile phase A consisted of 95% water, 5% methanol, 5 mM ammonium formate, and 0.1% formic acid, and mobile phase B was methanol/ACN (50:50, *v*/*v*). The final solution was transferred into an auto-filtering vial equipped with a 0.2 µm PTFE membrane filter for LC-MS/MS analysis.

### 2.4. LC-MS/MS Sample Analysis

Instrumental analysis was performed using a Shimadzu LCMS-8060NX triple quadrupole mass spectrometer (Shimadzu Corporation, Kyoto, Japan), coupled with an LC-40D XR pump, SIL-30AC autosampler, DGU-20A5R and DGU-405 degassing units, CBM-40 system controller, and CTO-20AC column oven. Chromatographic separation was achieved using an Accucore C18 column (2.1 × 100 mm, 2.6 µm; Thermo Scientific™, Waltham, MA, USA) maintained at 35 °C. Mobile phase A consisted of 95% water, 5% methanol, 5 mM ammonium formate, and 0.1% formic acid, and mobile phase B was composed of methanol/acetonitrile (50:50, *v*/*v*). The flow rate was optimized to 0.3 mL/min, and the injection volume was 10 µL. The gradient elution, starting at 5% phase B, held for 1 min, increased to 55% in 8.5 min, then ramped to 90% within 0.1 min and maintained for 2.9 min, was followed by a rapid return to 5% within 0.1 min, held constant until the end of analysis. The total run time was 15.5 min.

The mass spectrometer was operated using electrospray ionization (ESI) in negative mode, with a nebulizing gas flow of 3 L/min and a heating gas flow of 5 L/min. The interface temperature was maintained at 350 °C, while the desolvation line was at 180 °C, and the heat block at 300 °C. A drying gas flow of 10 L/min was applied to ensure optimal desolvation and ion transfer. These parameters were selected to maximize both sensitivity and stability during analysis. Table 1 summarizes the optimized multiple reaction monitoring (MRM) transitions for precursor and product ions, together with the corresponding LC-MS/MS parameters, including collision energy (CE), Q1 Pre Bias (voltage promotes the ionization of the precursor ion), and Q3 Pre Bias (voltage promotes the ionization of the product ion) for each analyte. Quantification was performed using the primary MRM transition (MRM 1), whereas the secondary transition (MRM 2) was used for confirmation and qualitative verification of each analyte.

### 2.5. Analytical Method Validation

Method validation was carried out in accordance with the SANTE/11312/2021 guidelines [26]. The linearity of the method was evaluated using four calibration curves prepared by spiking blank honey samples of different botanical origins (multifloral, chestnut, and acacia) with analytical standards of glyphosate, AMPA, and glufosinate ammonium at concentrations of 10, 20, 50, 100, and 300 µg/kg. Isotope-labeled internal standards specific for each analyte were used for procedural calibration and quantitative correction. The trueness and precision (expressed as repeatability RSDr) of the optimized method were assessed by spiking blank honey samples at two levels corresponding to LOQ and MRLs. Specifically, eight replicates were prepared at 10 µg/kg, and six replicates at 50 µg/kg were performed for each of the three different botanical origin honeys examined. This experimental design allowed the evaluation of matrix-related variability, which was minimized through the application of isotope-labeled internal standards and rigorous sample purification during the preparation phase. The LOQ, defined according to the SANTE guidance as the lowest concentration at which an analyte can be reliably quantified with acceptable accuracy and precision, was established at 10 µg/kg for each analyte. The MRLs for glyphosate and glufosinate in honey were both set at 0.05 mg/kg, in accordance with the Regulation (EC) No 396/2005 [14]. The SANTE/11312/2021document does not require the calculation or reporting of the limit of detection (LOD). As the method was specifically designed for routine laboratories to verify compliance with the EU MRLs for honey, determination of the LOD was considered unnecessary. The established LOQ of 10 µg/kg ensures reliable quantification well below the regulatory threshold, providing sufficient sensitivity for monitoring purposes. The matrix effect (ME), expressed as signal suppression or enhancement, was evaluated by comparing responses obtained from matrix addition and solvent-based standards, using the following formula [27]:

% ME = [(matrix signal intensity/solvent signal intensity) − 1] × 100

Finally, the overall performance of the method, including the suitability and optimization of each analytical step, was verified through participation in an international proficiency testing scheme.

### 2.6. Method Application

The validated method was applied to 126 honey samples of different botanical origins, predominantly multifloral, as this is the most commonly marketed type in Europe. All samples were produced in Italy and were either purchased specifically for this study or provided directly by beekeepers. Details regarding botanical and geographical origin are shown in Table S1 in the Supplementary Materials.

### 2.7. Statistical Analysis

Data analysis was performed using R software (version 4.3.1) [28]. Values below the limit of quantification (LOQ = 10 µg/kg) were treated as non-detects (0 µg/kg). Descriptive statistics were calculated for glyphosate concentrations, including frequency of detection, range, mean, and median. Differences in positivity rates (samples > LOQ) among geographical regions, macro-areas (North, Center, South/Islands), and botanical origins were evaluated using the chi-square (χ^2^) test for independence. The North area is constituted by Piedmont, Aosta Valley, Liguria, Lombardy, the Autonomous Province of Trento, the Autonomous Province of Bolzano, Veneto, Friuli Venezia Giulia, and Emilia-Romagna; the Center region is represented by Toscana, Umbria, Marche, Lazio, and Abruzzo, while the South/Island is constituted by Molise, Campania, Puglia, Basilicata, Calabria, Sicilia, and Sardinia.

Among positive samples, differences in glyphosate concentrations were assessed using the non-parametric Kruskal–Wallis test. A multivariable logistic regression model was applied to evaluate the association between glyphosate positivity (binary outcome) and predictor variables, including macro-area and honey type, using *Center* and *multifloral honey* as reference categories. Results were expressed as odds ratios (OR) with 95% confidence intervals (CI). Graphical representations (histograms and boxplots) were generated using matplotlib, and statistical significance was set at *p* < 0.05.

## 3. Results and Discussion

### 3.1. Method Development

**Figure 1:**
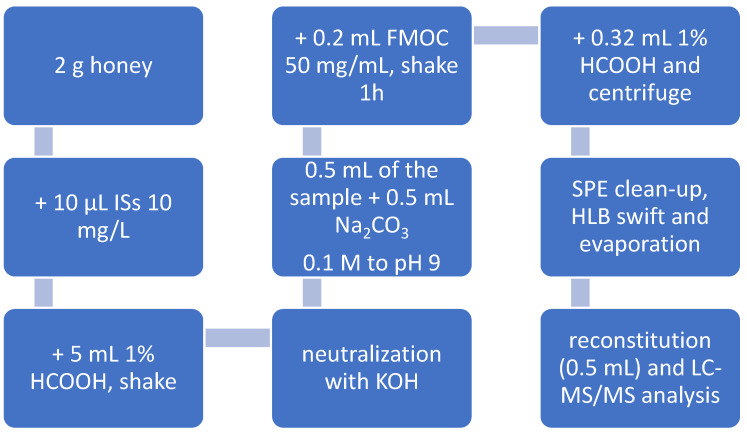
Honey preparation procedure for glyphosate, AMPA, and glufosinate ammonium analysis: The detailed sample preparation and extraction procedure is described in Section 2.3 of Section 2 (ISs: internal standards; HCOOH: formic acid; KOH: potassium hydroxide; Na_2_CO_3_: sodium carbonate; FMOC: fluorenylmethylchloroformate).

Initially, the method selected for the analysis of glyphosate, AMPA, and glufosinate ammonium in honey followed the procedure recommended by the EU Reference Laboratory for Single Residue Methods (EURL-SRM). This approach is based on the Quick Method for the Analysis of Highly Polar Pesticides in Food of Plant Origin, which involves extraction with acidified methanol, followed by LC- or IC-MS/MS analysis (QuPPe-PO Method, version 12.1, published in 2023 [29]). The QuPPe method offers the notable advantage of simple and rapid sample preparation, enabling the analysis of highly polar compounds in complex matrices such as honey. However, this benefit comes at the expense of limited sample clean-up, which leads to substantial matrix-related contamination of the mass spectrometer ion source and detector. After only a few chromatographic runs, such contamination results in a marked decline in analyte signal intensity, ultimately rendering the method unsuitable for routine laboratory use. To overcome this limitation, a sample preparation protocol was adopted for the quantification of glyphosate, AMPA, and glufosinate in honey based on FMOC derivatization, as described by Thompson et al. [25]. The derivatization process markedly reduces the polarity of the target analytes, thereby improving their retention on reversed-phase chromatographic columns and enhancing separation from matrix components. Furthermore, the introduction of hydrophobic moieties into the analyte structures increases their ionization efficiency in the ESI source, leading to improved LODs. The method was systematically optimized at each step and validated in-house. The final sample preparation and extraction procedure from honey is detailed in Section 2.3 (Section 2) and shown in Figure 1.

#### 3.1.1. Extraction Phase

Throughout the analytical workflow, plastic materials were used exclusively, as glyphosate is known to adhere to glass surfaces, potentially causing underestimation of its concentration and thereby introducing bias in the analysis.

Following a series of analytical tests, the sample size was set to 2 g of honey, which provided an optimal balance between sample representativeness and matrix manageability, reducing the amount of interfering components while maintaining sensitivity for trace-level determination. For this study, isotope-labeled internal standards of glyphosate, AMPA, and glufosinate were used to correct for potential analytical errors. A volume of 10 μL of 10 mg/L of mixed internal standard solution was added to each sample prior to extraction, resulting in a final concentration of 0.05 mg/L.

Acidified water was chosen as the dilution and extraction medium to improve analyte accessibility to the derivatizing agent. An acidic environment promotes the dissociation of analyte complexes, as multivalent cations and matrix components tend to interact with the amphoteric functional groups of glyphosate and related compounds, forming stable complexes. Initial trials using water acidified with 0.1% formic acid yielded significantly lower recoveries than those obtained with 5 mL of a 1% formic acid solution, which was ultimately adopted as the extraction solvent. Sonication in an ultrasonic bath further improved analyte recovery by disrupting potential glyphosate complexes with honey. Initially, the use of DCM along with acidified water was also tested for the extraction process to reduce matrix interference [27]. Following centrifugation, partial phase separation was observed in the DCM phase, while sugars, amino acids, and natural polar compounds remained in the aqueous phase. However, this approach resulted in low recoveries, likely due to the loss of analytes during their extraction from the organic solvent layer, and was therefore discarded.

#### 3.1.2. Derivatization Phase

After the extraction step, about 240 µL of KOH is added to each sample, and the pH is adjusted to neutrality. Derivatization with FMOC-Cl proceeds via a nucleophilic substitution reaction under alkaline conditions, targeting the amino group in the molecules of glyphosate, AMPA, and glufosinate. An alkaline environment (pH ~ 9) is therefore essential for efficient derivatization [30]. During the derivatization reaction, hydrochloric acid is generated, requiring a buffering system capable of maintaining stable alkaline conditions throughout the process. Initial trials using sodium tetraborate demonstrated insufficient buffering capacity to support efficient derivatization of the analytes within the honey matrix. Consequently, a 0.1 M sodium carbonate solution was adopted, which effectively stabilized the pH and enabled the complete derivatization reaction to proceed without complications (e.g., pH drift or precipitation issues). According to the literature, the derivatization of glyphosate, AMPA, and glufosinate is generally performed using relatively dilute solutions of FMOC-Cl in acetonitrile (ACN), ranging from 1.5 to 12 mg/mL, in matrices such as water, pollen, grape, and sucrose [27,31,32,33,34,35]. Thompson et al. [25] successfully derivatized these analytes in a honey matrix using a 50 mg/mL FMOC-Cl solution. In this study, FMOC-Cl concentrations of 5, 6, and 25 mg/mL were initially evaluated but proved insufficient to achieve complete derivatization. Because FMOC-Cl can also react with matrix constituents containing primary and secondary amino functional groups, it must be present in excess with respect to the analytes of interest [36]. *Apis mellifera* honey can contain up to 1% (*w*/*w*) free amino acids and 0.2–1.6% proteins [37], both of which can react with FMOC-Cl. However, excess FMOC-Cl can also react with water to form FMOC-OH, which can generate a visible precipitate in the presence of a high aqueous concentration of the medium. Therefore, the concentration and composition of the reaction medium (water–acetonitrile) must be carefully balanced to maintain both glyphosate and FMOC-Cl in solution while minimizing FMOC-OH formation. In this study, optimal conditions were achieved by adding 0.2 mL of 50 mg/mL FMOC-Cl to 1 mL of buffered sample, with continuous stirring to re-dissolve any precipitate.

Reported derivatization times for these analytes range from 30 min [27] to overnight incubation at room temperature in the dark [33]. In our tests, no significant differences were observed between the overnight and the one-hour reaction times, both conducted under gentle agitation. To ensure homogeneity and dissolve any precipitates, a three-minute vigorous agitation step was included at the start of the process. For efficiency and reproducibility, the one-hour protocol was adopted. Following incubation, the reaction was quenched by adding 0.32 mL of 1% formic acid, which reduced the pH to 4–5. This pH adjustment prior to the purification step improved analyte retention on the HLB cartridge.

#### 3.1.3. Clean-Up Phase

If injected directly, the sample solution containing carbonate buffer, unreacted FMOC-Cl, and FMOC-OH could create deposits in the curtain plate or in the capillary of the ESI source, resulting in decreased ionization efficiency and reduced response after only a few sample injections. To mitigate these issues, several purification strategies have been used to date, including simple partitioning with DCM [31,32], direct injection into an online solid phase extraction (SPE)-LC-MS/MS system [25,35], and the use of traditional HLB SPE cartridges [27,33]. In preliminary tests, DCM partitioning resulted in suboptimal recovery rates. To improve the purification process, we optimized the clean-up phase by employing HLB cartridges, which demonstrated enhanced performance. The derivatized solution, previously adjusted to pH 4–5, was loaded directly onto preconditioned cartridges (activated with methanol followed by 1% formic acid). The resin was subsequently washed with ultrapure water and DCM, dried under vacuum, and eluted with 5 mL of methanol. An alternative washing protocol using ethyl acetate, as proposed by Malaysiak et al. [33], was also evaluated; however, this approach yielded lower recovery and was therefore not adopted.

#### 3.1.4. Chromatographic Condition

**Figure 2:**
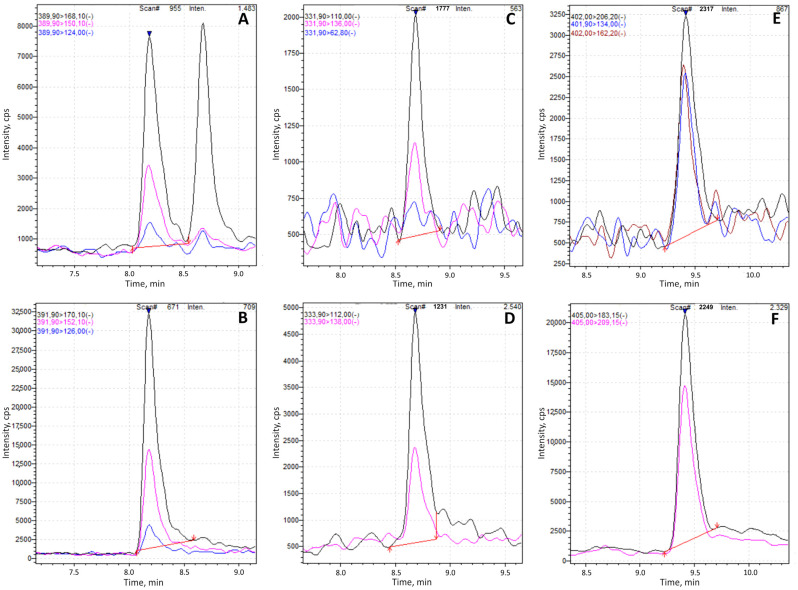
Multifloral honey spiked with glyphosate-FMOC (**A**), AMPA-FMOC (**C**), and glufosinate-FMOC (**E**) at the LOQ level of 10 µg/kg, along with their respective internal standards: glyphosate-2-^13^C,^15^N–FMOC (**B**), ^13^C-^15^N-AMPA-FMOC (**D**), and D_3_-glufosinate-FMOC (**F**), each added at 50 µg/kg.

FMOC-derivatized standards of glyphosate, AMPA, and glufosinate were employed to optimize chromatographic conditions. The optimization process included a systematic evaluation of different stationary phases, mobile phase compositions, and flow rates to achieve optimal separation and detection performance. The columns evaluated included Accucore aQ (2.1 × 100 mm, 2.6 µm; Thermo Scientific™) and Atlantis T3 (2.1 × 150 mm, 3 µm; Waters), tested using the EURL method. Additional evaluations were performed using Accucore C18 (2.1 × 100 mm, 2.6 µm; Thermo Scientific™, Waltham, MA, USA) and XTerra MS C18 (2.1 × 100 mm, 5 µm; Waters Corporation, Milford, MA, USA). Among these, the Accucore C18 column demonstrated superior chromatographic performance, characterized by enhanced peak areas and improved peak shape. Consequently, it was selected for the final method, which employed mobile phase A, consisting of 95% water, 5% methanol, 5 mM ammonium formate, and 0.1% formic acid, and mobile phase B, composed of methanol/ACN (50:50, *v*/*v*). Flow rate and gradient conditions were systematically adjusted to minimize peak tailing and enhance separation efficiency, ensuring accurate quantification of all target analytes within a reasonable total run time. These adjustments were validated through comparative evaluation of peak symmetry, area, and resolution across different conditions. Optimization of the organic mobile phase (B) and the gradient profile was required to address an isobaric interference observed in honey samples following FMOC derivatization. This interfering peak was identified and characterized using high-resolution liquid chromatography coupled to mass spectrometry (UHPLC Ultimate 3000 system interfaced with a Q-Exactive quadrupole-orbitrap mass spectrometer, Thermo Fisher Scientific). Initially, the interference co-eluted with both derivatized glyphosate and its corresponding internal standard, compromising quantification accuracy. Through systematic adjustment of chromatographic parameters, including mobile phase composition and gradient slope, baseline separation of the analyte and interfering peak was successfully achieved, resulting in excellent resolution and improved analytical reliability (Figure 2).

### 3.2. Method Validation Results

The analytical method was validated in accordance with the criteria outlined in the SANTE/11312/2021 guidance document on Analytical Quality Control and Method Validation Procedures for Pesticide Residues Analysis in Food and Feed [26]. All mandatory parameters, including sensitivity, linearity, mean recovery (trueness), precision (expressed as repeatability, RSDr), and LOQ, were systematically evaluated. In addition to quantitative performance characteristics, qualitative identification criteria, such as ion ratio and retention time, were also assessed. Validation experiments were conducted using at least six replicates at both the LOQ and a higher concentration corresponding to the target MRL. A summary of the validation results is shown in Table 2.

Based on prior experience with honey matrices, both botanical origin and color were identified as critical factors affecting analytical performance. Notably, significant signal suppression was observed in darker honeys, such as chestnut and honeydew, even for analytes like glyphosate, AMPA, and glufosinate (Figure S1 in the Supplementary Materials presents a comparative chromatographic profile of light versus dark honey matrices). To account for matrix variability and ensure method robustness, validation was performed using acacia, multifloral, and chestnut honeys, three of the most commercially relevant honey types in Europe. Sensitivity and linearity were assessed using matrix-matched calibration curves at concentrations of 10, 20, 50, 100, and 300 µg/kg, encompassing both the LOQ (10 µg/kg) and the MRL (50 µg/kg) established for glyphosate and glufosinate. Linearity was demonstrated with correlation coefficients (R^2^) ≥ 0.9967 for all analytes, and back-calculated concentrations deviated by no more than ±20% from nominal values, in compliance with the SANTE/11312/2021 acceptance criteria.

Matrix effect, exceeding 20% in terms of signal suppression, was initially assessed using commercially available derivatized analytical standards by comparing calibration curves prepared in solvent and in matrix. However, because both analytes and their isotope-labeled internal standards (not available in derivatized form commercially) required in-house derivatization, a direct comparison was not feasible in the final method. To mitigate potential matrix effects, analyte-specific internal standards and optimized sample preparation steps were employed, ensuring accurate quantification and method robustness. The LOQ was set at 10 µg/kg for glyphosate, AMPA, and glufosinate, and this concentration met the performance criteria established by the SANTE/11312/2021 guidance, as detailed in Table 1. Specificity was verified by analyzing blank honey samples (previously analyzed honey samples tested and confirmed negative for the target analytes) and solvent blanks processed in parallel. The responses from blank matrices and solvent controls did not exceed 30% of the signal corresponding to the LOQ, confirming the absence of significant interferences.

Recoveries, calculated as the mean across all spiking levels, were within the acceptable range of 70–120% as specified by the SANTE document. Method precision, expressed as RSDr, was calculated by dividing the standard deviation of replicate measurements at each spike level by the corresponding mean recovery and multiplying by 100. All RSDr values were below 20%, in compliance with validation requirements. Within-laboratory reproducibility (RSDwR ≤ 20%) and method robustness will be further assessed through quality control data generated during routine analyses, excluding variability attributable to sample heterogeneity.

Internal standards were added to each sample prior to the extraction and derivatization steps to monitor derivatization efficiency and to accurately assess analyte recovery. This approach also enabled the evaluation of matrix effects associated with different botanical origins of honey. Notably, darker honeys such as chestnut, honeydew, and certain multifloral varieties exhibited pronounced signal suppression compared to lighter honeys like acacia, citrus, or light multifloral types. Validation experiments conducted across a broad range of honey matrices, from light acacia to dark chestnut, confirmed that the use of analyte-specific internal standards effectively compensated for matrix-induced variability, maintaining quantitative accuracy regardless of matrix composition and color. Whenever possible, blank honey of the same botanical origin was used in analytical runs to minimize matrix mismatch; otherwise, a multifloral honey with intermediate color and no detectable residues was employed as a representative blank matrix.

During the method development phase, we participated in the EU Proficiency Test on the Analysis of Honey for Incurred and Spiked Residues of Pesticides Requiring Single Residue Methods (EUPT–SRM18, May/June 2023), organized by EURL-SRM. For glyphosate, the method yielded a Z-score of 1, indicating satisfactory performance and alignment with the assigned value. The proficiency test material was subsequently reanalyzed using the finalized method, confirming the accuracy of the procedure. These results align with the internal validation outcomes, further supporting the reliability and robustness of the method across different honey matrices and analytical conditions.

### 3.3. Real Samples Analysis

**Figure 3:**
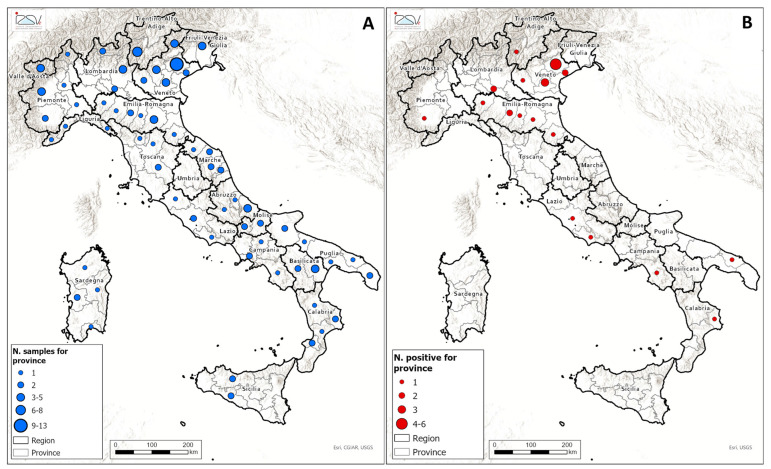
The spatial distribution of the 126 honey samples collected and analyzed across the Italian territory (**A**), and the corresponding locations of samples exhibiting quantifiable residues, defined as concentrations exceeding the established LOQ of 10 µg/kg (**B**).

A total of 126 honey samples produced in Italy were analyzed for the presence of glyphosate, AMPA, and glufosinate ammonium residues. The spatial distribution of the sample is illustrated in Figure 3A, while details regarding the botanical and geographical origin, as well as the analytes detected in the samples, are provided in Table S1 of the Supplementary Materials. The geographical distribution of the samples testing positive for glyphosate is illustrated in Figure 3B. Glyphosate residues above the LOQ were detected in 27 samples (21%), whereas concentrations exceeding the MRL were observed in 6 samples (5%). Detected glyphosate concentrations ranged from 10 to 519 µg/kg. No residues of AMPA or glufosinate ammonium were detected in any of the samples analyzed. The majority of the glyphosate-positive honey samples originated from Northern Italy, particularly from the Veneto and Emilia-Romagna regions. The spatial distribution of glyphosate residues in honey appears to reflect regional agrochemical usage patterns, especially in areas characterized by intensive conventional agriculture. According to the Italian National Institute of Statistics (ISTAT) data [38], the area of the Po Valley—including the Veneto, Emilia-Romagna, Lombardy, and parts of Piedmont regions—represents the core of Italy’s agricultural production. This area, known for its fertile soils, flat territory, abundant water resources, and advanced mechanization, accounts for approximately 35% of the national agricultural output. Veneto and Emilia-Romagna are particularly associated with high-input farming systems, where glyphosate can be applied as a pre-harvest desiccant or for weed control in crop rotation schemes. An example of a multifloral honey sample testing positive for glyphosate is shown in Figure S2.

The results of this study confirm the potential for glyphosate contamination in honey and are consistent with findings reported by other research groups. The scientific literature on glyphosate residues in honey remains limited, as highlighted in a recent review [15], which identified only 20 studies worldwide. Collectively, these studies analyzed 1965 honey samples, detecting glyphosate in 625 samples (32%), with reported concentrations ranging from 2 µg/kg to 5500 µg/kg. Since the publication of that review, five additional studies have investigated the presence of glyphosate, glufosinate, and related metabolites in honey [24,39,40,41,42], including two conducted specifically on Italian honey. The first analyzed 97 samples, detecting glyphosate in 12% of them, although none exceeded the MRL [42]. The second study involved a three-year monitoring campaign covering 221 samples, in which glyphosate was detected in 62 samples (28%), with 8 exceeding the MRL, though overall concentrations remained relatively low [39]. A recent EURL pilot monitoring project, which analyzed 187 honey samples from over 30 EU and non-EU countries, confirmed the presence of glyphosate residues in a notable proportion of samples, albeit generally below the EU MRL of 50 µg/kg, and emphasized the heterogeneity of analytical approaches among laboratories [43]. Targeted surveys conducted by New Zealand Food Safety in 2017–2019 on Manuka and other honey types detected glyphosate residues only in a small number of samples, all well below the MRLs set by New Zealand regulations [44]. Monitoring data from the U.S. Food and Drug Administration between 2016 and 2024 revealed glyphosate residues in all honey samples tested, including those labeled as organic, with concentrations occasionally reaching up to twice the EU MRL [45]. Overall, our results—obtained from a representative set of 126 honey samples—are consistent with both national and international studies, confirming the widespread but generally low-level occurrence of glyphosate residues in honey, with only isolated exceedances of regulatory limits.

Regarding AMPA, the review by Rampazzo et al. [15] reported its detection in 208 out of 471 honey samples (44%), with concentrations ranging from 1.9 to 100 µg/kg. In our dataset, AMPA was not detected in any of the 126 honey samples analyzed, despite the occurrence of glyphosate residues in several cases. This absence may indicate that glyphosate degradation to AMPA did not occur under the environmental or storage conditions relevant to our samples, or that AMPA concentrations were below the analytical method’s LOQ. The environmental transformation of glyphosate into AMPA is well documented in soil and water systems, where microbial activity plays a key role [46,47]. However, its occurrence within hive matrices remains poorly understood. The lack of AMPA detection in our samples precludes correlation analysis between glyphosate and its metabolite; nonetheless, future investigations employing lower LOQs or targeted studies on in-hive transformation mechanisms could provide valuable insights into potential metabolic or environmental degradation pathways.

Among the available literature, the study by Thompson et al. [25] diverges markedly from most published data, reporting the highest detection rates for glyphosate (99%), AMPA (99%), and glufosinate (62%) in 200 honey samples from Canada. This discrepancy reflects the lower LOQ applied in that study (1 µg/kg for each analyte), enabling the detection of trace levels not captured elsewhere. It also remains the only study to report glufosinate residues. Consistent with more recent investigations, neither AMPA nor glufosinate ammonium was detected in our dataset [39,40,41,42]. Only Sasano et al. [24] reported glufosinate in two of six samples purchased in Japan. In our study, no detectable glufosinate residues were found in any of the 126 Italian honey samples analyzed, confirming its very low occurrence in European honey. The low frequency of glufosinate detection may reflect its limited inclusion in monitoring programs (only eight studies to date), its lower global use compared to glyphosate, and the revocation of its EU approval in 2018 [48]. Nevertheless, its presence in honey cannot be completely ruled out, potentially arising from illegal use within the EU, contamination of imported honey from non-EU countries where glufosinate remains widely applied, or cross-border drift from neighboring regions. Given the significant proportion of annually imported honey into the EU market, such pathways represent plausible sources for occasional residues.

Collectively, our findings indicate that while Italian data align with European trends, showing moderate prevalence and generally low concentrations, global comparisons reveal substantial variability in detection rates and residue levels. These differences underscore the importance of harmonized monitoring protocols, comparable analytical sensitivity, and consistent reporting standards to ensure food safety, comparability of results across regions, reliable assessment of consumer exposure, and to support evidence-based risk management in apicultural products.

### 3.4. Statistical Analysis of Glyphosate Occurrence

**Figure 4:**
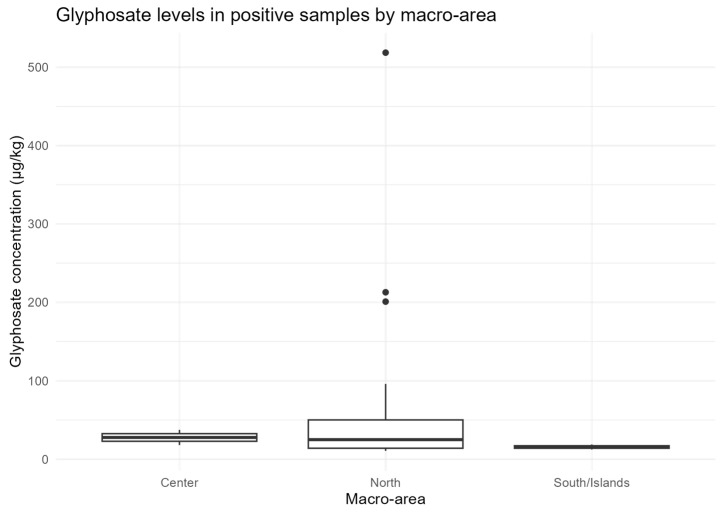
Glyphosate concentrations (µg/kg) in positive honey samples by macro-area in Italy. Boxplots show the distribution of glyphosate residues in honey samples exceeding the limit of quantification (LOQ = 10 µg/kg) across the three macro-areas (Center, North, South/Islands). Boxes represent the interquartile range (IQR), horizontal lines indicate medians, whiskers extend to 1.5 × IQR, and points denote outliers.

Overall, 27/126 samples (21.4%) were positive for glyphosate, with concentrations ranging from 10.7 to 518.7 µg/kg (median = 24.0 µg/kg; mean = 59.0 µg/kg). The proportion of glyphosate-positive samples differed significantly by macro-area (χ^2^(2) = 11.06, *p* = 0.004), with higher positivity in Northern Italy (23/72) compared to the Central (2/24) and Southern/Islands (2/30) regions. At the regional level, considering regions with ≥5 samples, the association remained significant (χ^2^(11) = 27.53, *p* = 0.0038), with the highest positivity observed in Veneto (12/27, 44%) and Emilia-Romagna (6/9, 67%), consistent with areas of intensive agriculture (Figure 4).

**Figure 5:**
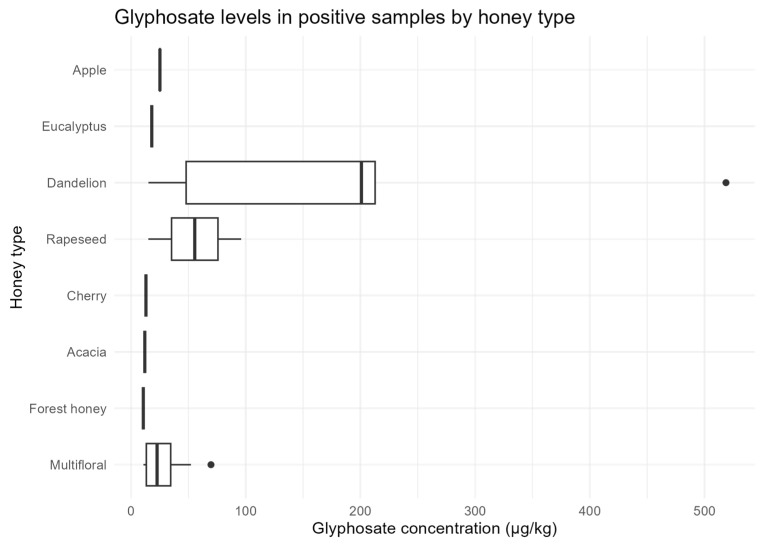
Glyphosate concentrations (µg/kg) in positive honey samples by botanical origin. Boxplots represent the distribution of glyphosate residues in honey samples exceeding the limit of quantification (LOQ = 10 µg/kg) across different botanical origins.

Positivity also differed significantly by botanical origin (χ^2^(3) = 23.06, *p* = 3.9 × 10^−5^). All dandelion honeys (5/5) and all apple honeys (2/2) tested positive. Dandelion samples showed the highest levels (median = 199 µg/kg; maximum = 518.7 µg/kg), whereas apple honey had a median concentration of 25 µg/kg. Multifloral honeys showed 14/88 positives (15.9%; median = 27 µg/kg), rapeseed 2/3 positives (median = 56 µg/kg), and acacia 1/5 (12 µg/kg). Cherry, eucalyptus, and forest honey exhibited only one positive sample each (Figure 5).

Dandelion and rapeseed honeys showed the highest concentrations, the widest variability, and the highest outlier (>500 µg/kg).

These results indicate a geographical pattern consistent with intensive conventional agriculture in the Po Valley, and suggest that botanical origin, particularly dandelion and apple, may influence glyphosate residue levels in honey, reflecting the phenology of flowering in areas of herbicide application.

## 4. Conclusions

The method developed and validated in this study enables the determination of glyphosate, its metabolite AMPA, and glufosinate in honey using LC-MS/MS instrumentation commonly available in routine analytical laboratories. The optimization and validation process demonstrated full compliance with the SANTE/11312/2021 criteria [26], confirming that the method achieves the required sensitivity, accuracy, and precision for reliable quantification of these highly polar pesticides. Developing robust and cost-effective analytical methods for such compounds remains a significant challenge, as they typically require advanced equipment such as ion chromatography or high-resolution mass spectrometry.

The method presented here overcomes these limitations, allowing for the determination of glyphosate, AMPA, and glufosinate in honey by utilizing the LC-MS/MS technique, an instrumentation commonly available in routine laboratories, and thus improving the accessibility of polar pesticide analysis in honey.

Considering the frequency and concentrations of glyphosate, AMPA, and glufosinate detected, our findings indicate that the low-level environmental glyphosate contamination represents a greater ecological concern for pollinators’ health than for human consumers. This is due to the harmful effects caused by widespread, low-concentration exposure to glyphosate, particularly under chronic exposure conditions. Although the concentrations observed were generally below regulatory limits for human consumption, chronic exposure to glyphosate, even at low levels, has been associated with sublethal effects on honey bees, including impaired navigation, reduced foraging efficiency, and suppressed immune responses. These effects can compromise colony health and productivity over time. From a consumer safety perspective, the detected concentrations do not indicate an immediate health risk, as all values remain well below the EU MRL. Nevertheless, the presence of glyphosate in honey highlights its pervasive environmental distribution and underscores the need for continued surveillance to ensure compliance and maintain consumer confidence in honey quality. The detection, or confirmed absence, of potentially hazardous substances in honey provides valuable insights for both food safety assurance and environmental risk assessment. This analytical approach contributes to improving the overall quality and traceability of beekeeping products, while also informing beekeepers and consumers about possible exposure sources, such as herbicide use near foraging areas. As emphasized by the EC, there is a pressing need to expand monitoring programs and increase the number of laboratories equipped to analyze these compounds in honey.

Wider implementation of validated methods such as the one described in this paper would strengthen the EU’s surveillance capacity, enhance data comparability, and ultimately support evidence-based strategies for both food safety and pollinator protection.

## Figures and Tables

**Table 1:** LC-MS/MS method parameters and MRM transitions for the quantification of target analytes and their corresponding internal standards.

Compound	Precursor *(m/z)*	Product Ions *(m/z)*	Q1 Pre Bias (V)	CE (eV)	Q3 Pre Bias (V)
Glyphosate-FMOC	389.9	MRM1 168.1 MRM2 150.1	−23.0 −24.0	−13.0 −24.0	−27.0 −29.0
AMPA-FMOC	331.9	MRM1 110.0 MRM2 136.0	−20.0 −20.0	−10.0 −16.0	−18.0 −23.0
Glufosinate ammonium-FMOC	402.0	MRM1 206.2 MRM2 162.2	−14.0 −26.0	−14.0 −24.0	−12.0 −26.0
Glyphosate-2-^13^C^15^N-FMOC	391.9	MRM1 170.1 MRM2 152.1	−25.0 −25.0	−13.0 −22.0	−30.0 −30.0
AMPA ^13^C^15^N-FMOC	333.9	MRM1 112.0 MRM2 138.0	−22.0 −12.0	−13.0 −15.0	−18.0 −25.0
Glufosinate-D_3_	405.0	MRM1 183.2 MRM2 209.2	−14.0 −26.0	−13.0 −16.0	−11.0 −12.0

Notes: CE: collision energy; Q1 Pre Bias: voltage promotes the ionization of the precursor ion; Q3 Pre Bias: voltage promotes the ionization of the product ion; MRM 1: Primary MRM transition; MRM 2: Secondary MRM transition.

**Table 2:** Method validation results for glyphosate, AMPA, and glufosinate in honey.

Analyte	Linearity Range (µg/L)	R^2^	Spiking Level (µg/kg)	Recovery (%)	RSDr (%)
Glyphosate-FMOC	10–300	0.9989	10	77	6
			50	104	9
AMPA-FMOC	10–300	0.9967	10	108	13
			50	99	11
Glufosinate ammonium-FMOC	10–300	0.9994	10	99	12
			50	99	10

## Data Availability

The original contributions presented in this study are included in the article/Supplementary Material. Further inquiries can be directed to the corresponding author.

## Supplementary Materials

The following supporting information can be downloaded at: https://www.mdpi.com/article/10.3390/foods14234050/s1, Figure S1: Comparative chromatographic profiles of light (acacia, panel A–F) and dark (chestnut, panel G–N) honey matrices spiked at 50 µg/kg (A,G glyphosate, C,I AMPA and E,M glufosinate, and B,H D,L, F,N their respective internal standards). Chromatographic profile showing retention time (min) on the x-axis and signal intensity (cps) on the y-axis; Figure S2: Multiflower honey analyzed for glyphosate (A), AMPA (C), and glufosinate (E) with their respective internal standards: glyphosate-2-13C,15N–FMOC (B), 13C-15N-AMPA-FMOC (D), and D3-glufosinate-FMOC (F), each added at 50 µg/kg. The sample resulted positive for glyphosate (12 µg/kg). Chromatographic profile showing retention time (min) on the x-axis and signal intensity (cps) on the y-axis; Table S1: Botanical and geographical origins of the Italian samples analyzed, and concentrations of analytes detected.

## Abbreviations

The following abbreviations are used in this manuscript:

ACN	Acetonitrile
AMPA	Aminomethylphosphonic Acid
DCM	Dichloromethane
DMSO	Dimethyl Sulfoxide
EFSA	European Food Safety Authority
EPA	United States Environmental Protection Agency
ESI	Electrospray Ionization
EU	European Union
EURL-SRM	EU Reference Laboratory for Single Residue Methods
FAO	Food and Agriculture Organization of the United Nations
FMOC-Cl	9-Fluorenylmethyl Chloroformate
GBHs	Glyphosate-Based Herbicides
HCOOH	Formic Acid
HLB	Hydrophilic–Lipophilic Balanced
IARC	International Agency for Research on Cancer
ISs	Internal Standards
ISTAT	Italian National Institute of Statistics
KOH	Potassium Hydroxide
LC-MS/MS	Liquid Chromatography Coupled with Tandem Mass Spectrometry
LOQ	Limit of Quantification
MACP	Multiannual Control Programs for Pesticide Residues
ME	Matrix Effect
MRLs	Maximum Residue Levels
MRM	Multiple Reaction Monitoring
Na_2_CO_3_	Sodium Carbonate
PAN	Pesticide Action Network
PCNs	National Control Programs
QuPPe-PO	Quick Method for the Analysis of Highly Polar Pesticides in Food of Plant Origin
RSDr	Relative Standard Deviation of Repeatability
SPE	Solid-Phase Extraction
WD	Pesticides Working Document
WHO	World Health Organization
