# Supplementary material for: The Development and Validation of an LC-MS/MS Method for the Determination of Glyphosate, AMPA, and Glufosinate in Honey Following FMOC-Cl Derivatization: Application to Italian Samples
Source: Foods. 2025 Nov 26;14(23):4050. doi: 10.3390/foods14234050 (PMC12692283; doi:10.3390/foods14234050)
Supplement: Supplementary file 1 [file foods-14-04050-s001.zip › foods-3971602-supplementary.pdf]

Development and Validation of an LC-MS/MS Method for the Determination of Glyphosate, AMPA, and Glufosinate in Honey Following FMOC-Cl Derivatization: Application to Italian Samples

Supplementary materials

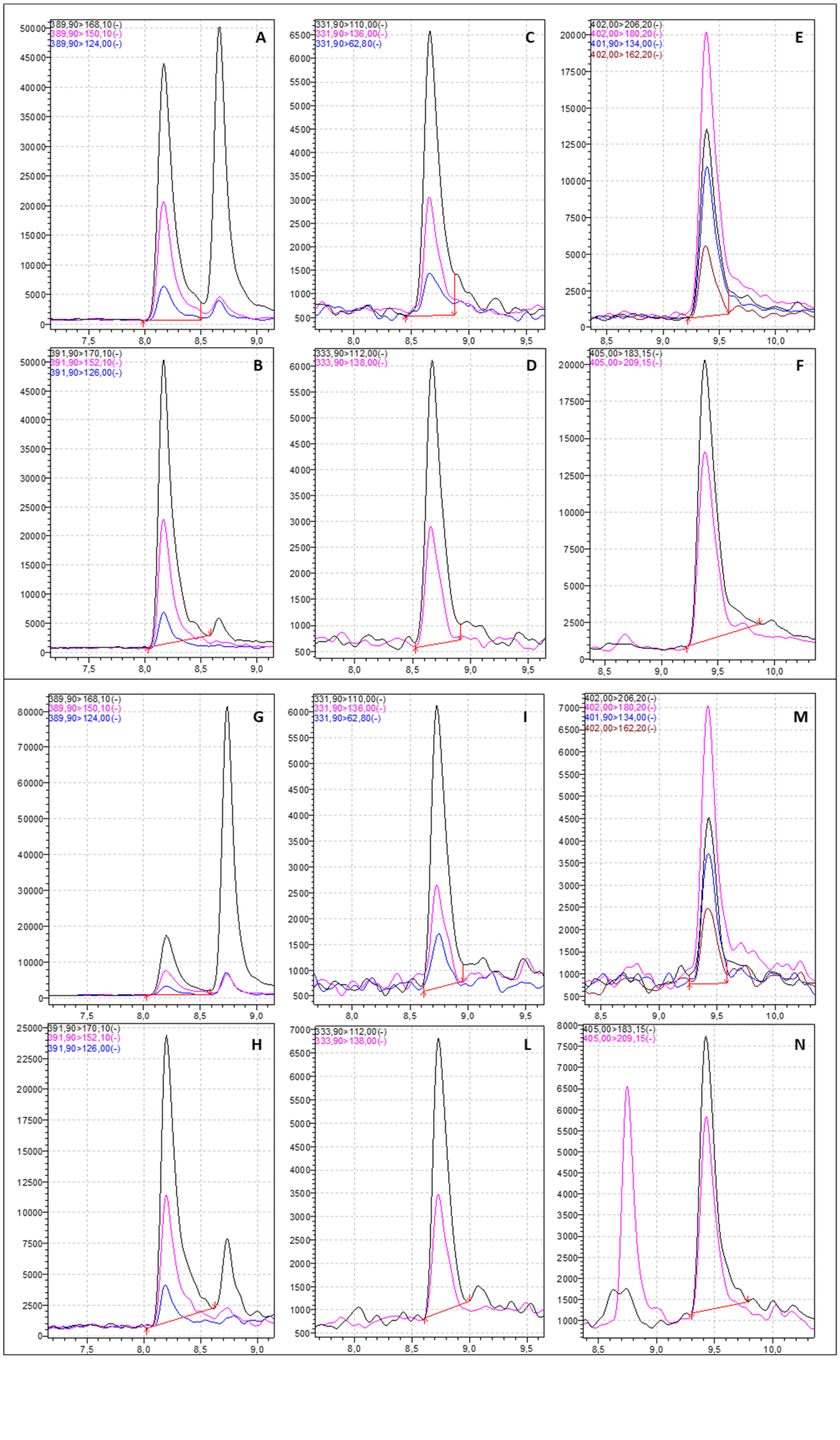

Figure S1: Comparative chromatographic profiles of light (acacia, panel A-F) and dark (chestnut, panel G-N) honey matrices spiked at 50 µg/kg (A,G glyphosate, C,I AMPA and E,M glufosinate, and B,H D,L, F,N their respective internal standards). Chromatographic profile showing retention time (min) on the x-axis and signal intensity (cps) on the y-axis.

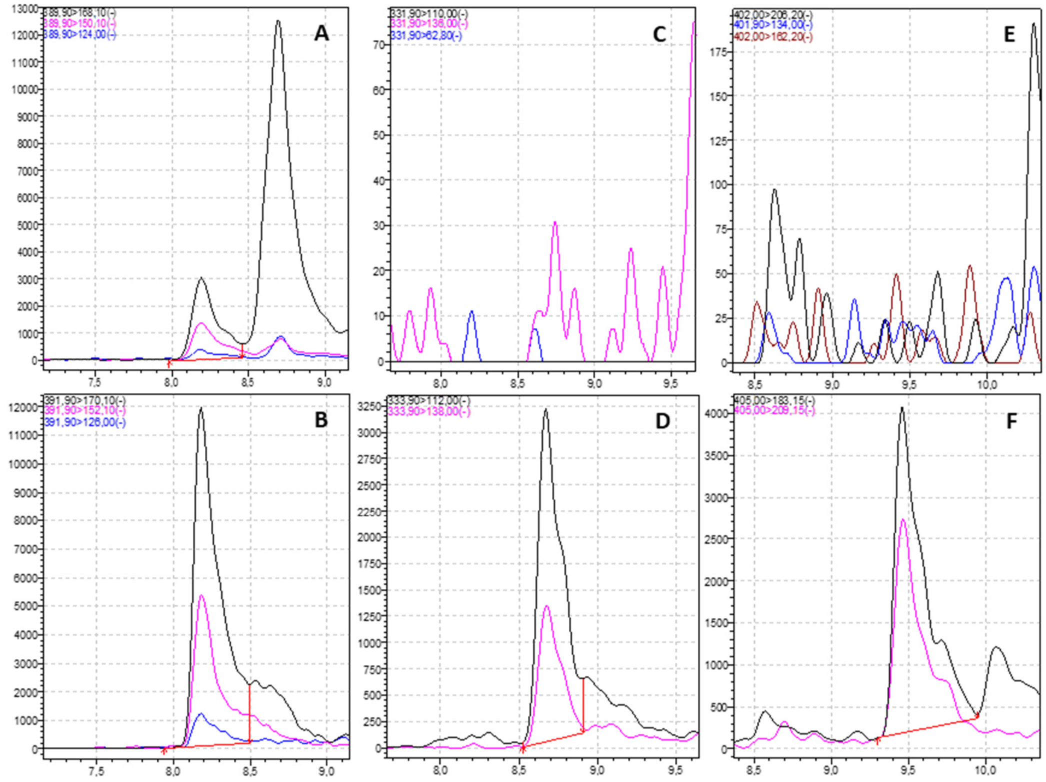

Figure S2: Multiflower honey analysed for glyphosate (A), AMPA (C), and glufosinate (E) with their respective internal standards: glyphosate-2-^{13}C,^{15}N–FMOC (B), ^{13}C-^{15}N-AMPA-FMOC (D), and D_3-glufosinate-FMOC (F), each added at 50 µg/kg. The sample resulted positive for glyphosate (12 µg/kg). Chromatographic profile showing retention time (min) on the x-axis and signal intensity (cps) on the y-axis.

Table S1: Botanical and geographical origins of the Italian samples analyzed, and concentrations of analytes detected.

Sample nr.	Botanical origin	Province	Region	glyphosate	AMPA	glufosinate
1	acacia	Treviso	Veneto	< LOQ	n.d.	n.d
2	acacia	Treviso	Veneto	n.d	n.d.	n.d
3	acacia	Venezia	Veneto	12	n.d.	n.d
4	acacia	Vicenza	Veneto	< LOQ	n.d.	n.d
5	acacia	Vicenza	Veneto	< LOQ	n.d.	n.d
6	citrus	Cosenza	Calabria	< LOQ	n.d.	n.d
7	citrus	Napoli	Campania	n.d	n.d.	n.d
8	citrus	Taranto	Puglia	< LOQ	n.d.	n.d

9	chestnut	Torino	Piemonte	n.d	n.d.	n.d
10	chestnut	Treviso	Veneto	n.d	n.d.	n.d
11	chestnut	Treviso	Veneto	n.d	n.d.	n.d
12	chestnut	Treviso	Veneto	n.d	n.d.	n.d
13	chestnut	Treviso	Veneto	n.d	n.d.	n.d
14	chestnut	Treviso	Veneto	n.d	n.d.	n.d
15	cherry	Treviso	Veneto	13	n.d.	n.d
16	rapeseed	Cremona	Lombardia	96	n.d.	n.d
17	rapeseed	Ancona	Marche	n.d	n.d.	n.d
18	rapeseed	Venezia	Veneto	15	n.d.	n.d
19	eucalyptus	Matera	Basilicata	n.d	n.d.	n.d
20	eucalyptus	Latina	Lazio	18	n.d.	n.d
21	eucalyptus	Oristano	Sardegna	n.d	n.d.	n.d
22	sunflower	Fermo	Marche	n.d	n.d.	n.d
23	sunflower	Macerata	Marche	n.d	n.d.	n.d
24	lemon	Vicenza	Veneto	< LOQ	n.d.	n.d
25	apple	Trento	Trentino-Alto Adige	25	n.d.	n.d
26	apple	Verona	Veneto	25	n.d.	n.d
27	multiflower	Chieti	Abruzzo	n.d	n.d.	n.d
28	multiflower	Chieti	Abruzzo	n.d	n.d.	n.d
29	multiflower	L'Aquila	Abruzzo	n.d	n.d.	n.d
30	multiflower	Pescara	Abruzzo	n.d	n.d.	n.d
31	multiflower	Matera	Basilicata	n.d	n.d.	n.d
32	multiflower	Matera	Basilicata	< LOQ	n.d.	n.d
33	multiflower	Potenza	Basilicata	n.d	n.d.	n.d
34	multiflower	Potenza	Basilicata	< LOQ	n.d.	n.d
35	multiflower	Catanzaro	Calabria	n.d	n.d.	n.d
36	multiflower	Crotone	Calabria	n.d	n.d.	n.d
37	multiflower	Crotone	Calabria	12	n.d.	n.d
38	multiflower	Vibo Valentia	Calabria	n.d	n.d.	n.d
39	multiflower	Vibo Valentia	Calabria	n.d	n.d.	n.d
40	multiflower	Benevento	Campania	< LOQ	n.d.	n.d
41	multiflower	Napoli	Campania	< LOQ	n.d.	n.d
42	multiflower	Salerno	Campania	19	n.d.	n.d
43	multiflower	Forlì Cesena	Emilia-Romagna	15	n.d.	n.d
44	multiflower	Modena	Emilia-Romagna	12	n.d.	n.d
45	multiflower	Parma	Emilia-Romagna	n.d	n.d.	n.d
46	multiflower	Piacenza	Emilia-Romagna	26	n.d.	n.d
47	multiflower	Reggio Emilia	Emilia-Romagna	52	n.d.	n.d
48	multiflower	Udine	Friuli Venezia Giulia	n.d	n.d.	n.d
49	multiflower	Udine	Friuli Venezia Giulia	n.d	n.d.	n.d
50	multiflower	Udine	Friuli Venezia Giulia	n.d	n.d.	n.d
51	multiflower	Udine	Friuli Venezia Giulia	n.d	n.d.	n.d
52	multiflower	Roma	Lazio	< LOQ	n.d.	n.d
53	multiflower	Roma	Lazio	38	n.d.	n.d

54	multiflower	Viterbo	Lazio	n.d	n.d.	n.d
55	multiflower	Imperia	Liguria	< LOQ	n.d.	n.d
56	multiflower	La Spezia	Liguria	n.d	n.d.	n.d
57	multiflower	Savona	Liguria	n.d	n.d.	n.d
58	multiflower	Brescia	Lombardia	n.d	n.d.	n.d
59	multiflower	Brescia	Lombardia	< LOQ	n.d.	n.d
60	multiflower	Brescia	Lombardia	< LOQ	n.d.	n.d
61	multiflower	Brindisi	Lombardia	11	n.d.	n.d
62	multiflower	Sondrio	Lombardia	n.d	n.d.	n.d
63	multiflower	Sondrio	Lombardia	n.d	n.d.	n.d
64	multiflower	Ancona	Marche	< LOQ	n.d.	n.d
65	multiflower	Fermo	Marche	< LOQ	n.d.	n.d
66	multiflower	Macerata	Marche	n.d	n.d.	n.d
67	multiflower	Pesaro e Urbino	Marche	n.d	n.d.	n.d
68	multiflower	Campobasso	Molise	n.d	n.d.	n.d
69	multiflower	Campobasso	Molise	n.d	n.d.	n.d
70	multiflower	Isernia	Molise	n.d	n.d.	n.d
71	multiflower	Isernia	Molise	< LOQ	n.d.	n.d
72	multiflower	Cuneo	Piemonte	n.d	n.d.	n.d
73	multiflower	Torino	Piemonte	n.d	n.d.	n.d
74	multiflower	Torino	Piemonte	n.d	n.d.	n.d
75	multiflower	Verbania	Piemonte	< LOQ	n.d.	n.d
76	multiflower	Vercelli	Piemonte	n.d	n.d.	n.d
77	multiflower	Foggia	Puglia	n.d	n.d.	n.d
78	multiflower	Foggia	Puglia	n.d	n.d.	n.d
79	multiflower	Lecce	Puglia	n.d	n.d.	n.d
80	multiflower	Lecce	Puglia	n.d	n.d.	n.d
81	multiflower	Nuoro	Sardegna	n.d	n.d.	n.d
82	multiflower	Oristano	Sardegna	n.d	n.d.	n.d
83	multiflower	Sassari	Sardegna	n.d	n.d.	n.d
84	multiflower	Agrigento	Sicilia	< LOQ	n.d.	n.d
85	multiflower	Agrigento	Sicilia	< LOQ	n.d.	n.d
86	multiflower	Palermo	Sicilia	n.d	n.d.	n.d
87	multiflower	Palermo	Sicilia	n.d	n.d.	n.d
88	multiflower	Firenze	Toscana	n.d	n.d.	n.d
89	multiflower	Pistoia	Toscana	n.d	n.d.	n.d
90	multiflower	Siena	Toscana	n.d	n.d.	n.d
91	multiflower	Siena	Toscana	n.d	n.d.	n.d
92	multiflower	Trento	Trentino-Alto Adige	n.d	n.d.	n.d
93	multiflower	Trento	Trentino-Alto Adige	n.d	n.d.	n.d
94	multiflower	Trento	Trentino-Alto Adige	n.d	n.d.	n.d
95	multiflower	Trento	Trentino-Alto Adige	n.d	n.d.	n.d
96	multiflower	Trento	Trentino-Alto Adige	n.d	n.d.	n.d
97	multiflower	Trento	Trentino-Alto Adige	n.d	n.d.	n.d

98	multiflower	Trento	Trentino-Alto Adige	< LOQ	n.d.	n.d
99	multiflower	Aosta	Valle d'Aosta	< LOQ	n.d.	n.d
100	multiflower	Aosta	Valle d'Aosta	n.d	n.d.	n.d
101	multiflower	Aosta	Valle d'Aosta	n.d	n.d.	n.d
102	multiflower	Aosta	Valle d'Aosta	n.d	n.d.	n.d
103	multiflower	Belluno	Veneto	n.d	n.d.	n.d
104	multiflower	Belluno	Veneto	n.d	n.d.	n.d
105	multiflower	Belluno	Veneto	n.d	n.d.	n.d
106	multiflower	Padova	Veneto	13	n.d.	n.d
107	multiflower	Padova	Veneto	24	n.d.	n.d
108	multiflower	Padova	Veneto	70	n.d.	n.d
109	multiflower	Treviso	Veneto	21	n.d.	n.d
110	multiflower	Treviso	Veneto	25	n.d.	n.d
111	multiflower	Treviso	Veneto	45	n.d.	n.d
112	multiflower	Vicenza	Veneto	n.d.	n.d.	n.d
113	multiflower	Verona	Veneto	< LOQ	n.d.	n.d
114	forest honey	Bologna	Emilia-Romagna	11	n.d.	n.d
115	multiflower	Chieti	Abruzzo	n.d	n.d.	n.d
116	rhododendron	Aosta	Valle d'Aosta	n.d	n.d.	n.d
117	blackberry	Imola	Emilia-Romagna	< LOQ	n.d.	n.d
118	dandelion	Reggio Emilia	Emilia-Romagna	48	n.d.	n.d
119	dandelion	Cremona	Lombardia	201	n.d.	n.d
120	dandelion	Cuneo	Piemonte	15	n.d.	n.d
121	dandelion	Treviso	Veneto	213	n.d.	n.d
122	dandelion	Treviso	Veneto	519	n.d.	n.d
123	lime	Bologna	Emilia-Romagna	n.d	n.d.	n.d
124	lime	Alessandria	Piemonte	< LOQ	n.d.	n.d
125	clover	Barletta-Andria-Trani	Puglia	n.d	n.d.	n.d
126	clover	Cagliari	Sardegna	n.d	n.d.	n.d
